# Overcoming challenges for designing and implementing the One Health approach: A systematic review of the literature

**DOI:** 10.1016/j.onehlt.2019.100085

**Published:** 2019-03-18

**Authors:** Carolina dos S. Ribeiro, Linda H.M. van de Burgwal, Barbara J. Regeer

**Affiliations:** a The Netherlands National Institute for Public Health and the Environment (RIVM), Center for Infectious Disease Control, Bilthoven, Netherlands; b Athena Institute for Research on Innovation and Communication in Health and Life Sciences, Vrije Universiteit Amsterdam, Amsterdam, Netherlands

**Keywords:** One health, Challenges, Design, Implementation, Interdisciplinary collaboration, Transdisciplinary research, strategic solutions, EIDs, Emerging infectious diseases, ID, Interdisciplinary, OH, One Health, TD, Transdisciplinary

## Abstract

Collaborative approaches in health, such as One Health (OH), are promising; nevertheless, several authors point at persistent challenges for designing and implementing OH initiatives. Among other challenges, OH practitioners struggle in their efforts to collaborate across disciplines and domains. This paper aims to provide insights into the existing challenges for designing and implementing OH initiatives, their causes and solutions, and points out strategic solutions with the potential to solve practical challenges. A systematic literature search was performed for emerging challenges and proposed solutions in the process of conducting OH initiatives. Next, a thematic and a causal analysis were performed to unravel challenges and their causes. Finally, solutions were discriminated on whether they were only recommended, or implemented as a proof-of-principle. The 56 included papers describe 21 challenges endured by OH initiatives that relate to different themes (policy and funding; education and training; surveillance; multi-actor, multi-domain, and multi-level collaborations; and evidence). These challenges occur in three different phases: the acquisition of sufficient conditions to start an initiative, its execution, and its monitoring and evaluation. The findings indicate that individual challenges share overlapping causes and crosscutting causal relations. Accordingly, solutions for the successful performance of OH initiatives should be implemented to tackle simultaneously different types of challenges occurring in different phases. Still, promoting collaboration between the wide diversity of stakeholders, as a fundamental aspect in the OH approach, is still by far the most challenging factor in performing OH initiatives. Causes for that are the difficulties in promoting meaningful and equal participation from diverse actors. Solutions proposed for this challenge focused on guiding stakeholders to think and collaborate beyond their professional and cultural silos to generate knowledge co-creation and innovative methodologies and frameworks. Finally, the biggest knowledge gap identified, in terms of proposed solutions, was for monitoring and evaluating OH initiatives. This highlights the need for future research on evaluation methods and tools specific for the OH approach, to provide credible evidence on its added value. When considering challenges endured by former OH initiatives and the proposed solutions for these challenges, practitioners should be able to plan and structure such initiatives in a more successful way, through the strategic pre-consideration of solutions or simply by avoiding known barriers.

## 1 Introduction

During the 21st century, diverse global health problems have emerged, mostly related to climate change, environmental sustainability, zoonotic and (re)emerging infectious diseases (EIDs). Innovative actions are needed to effectively address such problems. Defined as “a worldwide strategy for expanding interdisciplinary collaborations and communications in all aspects of health for people, animals and the environment” [1], One Health (OH) initiatives are promising. In fact, the OH approach is not a new concept; for many years, the link between animal and human health has been recognized. Nevertheless, it has suffered constant modifications in its definition and scope. From One Medicine, aimed at addressing animal-human interactions; passing through Ecohealth, advocating for the inclusion of the environmental components of health; to One World – One Health, the OH approach has finally recognized the importance of linking human, animal and environmental health [[2], [3], [4], [5], [6]].

Still, several authors have mentioned persistent challenges for designing and implementing OH initiatives, many of which are related to the necessity to include a wide range of relevant professionals, since each contribute valuable skills and perspectives that will ultimately advance the cause [7]. Despite the consensual OH definition that emerged in recent years, the level of collaboration required in the OH approach is not yet agreed upon. Most of the OH initiatives are based on interdisciplinary (ID) collaborations, implying the integration of different disciplines and cooperation between diverse experts, creating possibilities for knowledge co-creation. More recent initiatives, however, advocate for taking a whole society approach, which implies a transdisciplinary (TD) level of collaboration, with the inclusion of stakeholders beyond the academic domain [8,9]. Some authors advocate that taking a TD approach can better address complex global health challenges, due to the consideration of local contexts and the inclusion of community stakeholders, which contribute to the adoption and sustainability of OH initiatives [8,9].

The inclusion of diverse stakeholders under the OH approach, however, can lead to conflicts due to their manifold interests and priorities. Examples of collaborative conflicts are power imbalances, conflicts of interest and coordination gaps that can occur at an ID level but especially at a cross-sectoral, participatory TD level [10]. Besides these collaboration barriers, the different interpretations of the OH scope translate into diversity in the implementation of OH initiatives, generating challenges for the development of standards and guidelines for OH practitioners. This empirical gap and the manifold challenges endured by OH initiatives have been identified in the literature [2,4,11], highlighting the need for guidance on how to address collaboration and implementation challenges for the OH approach.

Reviewing and understanding such challenges is therefore essential to address the complexity in performing OH initiatives. Zoonotic and emerging infectious diseases require fast actions that are only possible through the design of OH initiatives in line with the acknowledgement of potential challenges and possible solutions [12]. Through a systematic literature review, this paper identifies challenges for designing and implementing OH initiatives, their causes and proposed solutions. The objective is to support the development and performance of the OH approach by providing insights on existing challenges, besides identifying proposed and implemented solutions that have the potential to solve multiple challenges.

## 2 Materials and methods

A systematic review of the literature was carried out to identify potential challenges and possible solutions for designing and implementing OH initiatives.

### 2.1 Search strategy

In order to provide an overview of challenges to designing and implementing OH initiatives and enable further discussion on how those initiatives attempt to address such challenges, we sampled the population of published peer-reviewed papers that mentioned OH initiatives and the challenges endured by them. For the systematic search, the online databases of PubMed and Web of Science were used, due to their broad scope of publication and multi-disciplinary contents. The key terms applied on the search were “one health” and “challenges”. The final searching syntax was as the following: (challenges OR difficulties OR barriers OR problems) AND (“one health” OR “eco health” OR “one medicine”). The final search was performed on August 31, 2017.

### 2.2 Eligibility criteria and selection of studies

**Fig. 1:**
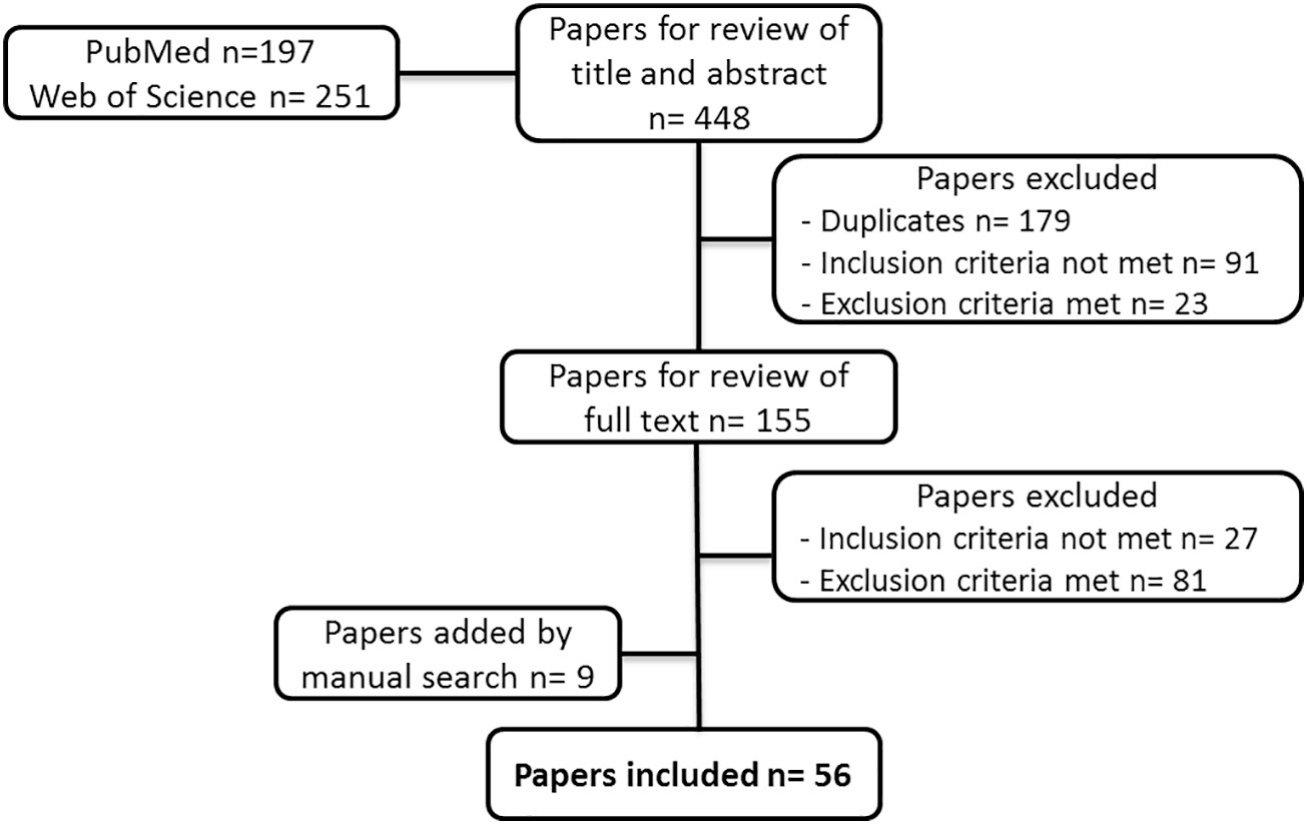
Selection of papers. After employing the search syntax and the selection process based on inclusion and exclusion criteria, 56 papers were included in this review.

After the systematic search, different inclusion and exclusion criteria were used to select the papers for this review. Papers were included when they considered OH initiatives according to the definition and scope applied by the OH initiative movement [1]. This scope consists of two aspects: 1) aiming to enhance collaboration and communication in all aspects of health and 2) recognizing the link between animal, human and/or environmental domains. Papers were excluded when they described OH as an outcome (e.g. did not discuss challenges endured by OH initiatives, but the challenges that lead to their creation), or when they did not present any challenges. The search was not restricted by year of publication, study design or any further factors, but language (English). Fig. 1 depicts the selection process for the inclusion and exclusion of papers.

After removing duplicates that were identified by both databases, the papers were screened in two phases: first the title and abstract, and second the full text of the papers were screened. Papers were excluded if they did not meet the inclusion criteria by not referring to OH initiatives, or by employing a OH definition that was not in accordance with the definition and scope previously described (e.g. discussed challenges from a single disciplinary perspective not engaging in cross-disciplinary collaborations). Additionally, papers that did not discuss empirical challenges endured by OH initiatives or discussed challenges that lead to their creation (OH as an outcome) were excluded. After conducting the systematic search, the authors identified extra articles of relevance to the topic of this review; those were added manually to the dataset. A total of 56 papers were included in this review, which were diverse in type of study, geographic location, approach, and field of publication (see Table A.1 in the Appendix). The included articles were compiled for analysis with the aid of the referencing software Endnote.

### 2.3 Data extraction and analysis

After the selection of studies, papers were screened systematically to identify challenges and proposed solutions within the breadth of the literature. Secondly, a thematic analysis was performed to classify the identified challenges and solutions following both chronological phases of realization and emerging themes. Solutions were classified on their extent of implementation indicating whether they were only recommended or also implemented in specific cases. Thirdly, a causal analysis was conducted, through the construction of a causal tree, to unravel arguments for defining causal relations between challenges [[13], [14], [15]]. Finally, a semi-quantitative analysis was performed to measure the frequency in which challenges and solutions were mentioned by the included papers. The authors highlight here that although the search strategy was focused on identifying challenges in the performance of One Health initiatives, most of the included papers either proposed or discussed solutions to address such challenges, which are later elaborated in this review by discriminating practical examples of implementation.

## 3 Results

### 3.1 Defining OH challenges

From the analysis of the 56 included papers, 21 single challenges for designing and implementing OH initiatives were identified. Based on the thematic analysis, the challenges were classified in different themes that emerged during the data analysis. Firstly, challenges were grouped in three distinct chronological process phases: (a) conditions for starting, (b) execution, and (c) monitoring and evaluation. Although chronological process phases were used to group challenges, a pertinent point is that in practice such phases are not necessarily consecutive and unrelated, but rather interactive and iterative [16]. In other words, phases can and usually do occur in parallel mode, overlapping from time to time. Secondly, challenges were classified in the emerging themes of policy and funding, education and training, surveillance, multi-actor (ID) collaborations, multi-domain (TD) collaborations, multi-level collaborations (across different institutional levels), and evidence.

#### 3.1.1 Conditions for starting

Challenges under this group relate to difficulties for OH practitioners to acquire and establish the necessary conditions for starting their initiatives. Conditions for starting and executing projects have to be created at different levels: at an external and institutional context level (systemic); and at an internal local context within projects [16]. Still, the acquisition of conditions has to be performed not only in the beginning of a project but throughout the whole process (including execution and monitoring and evaluation). In relation to the thematic classification, challenges for acquiring conditions for starting OH initiatives comprise the themes of insufficient policy prioritization and funding, and the lack of OH educational and training programs, as described in Table 1.

**Table 1:** Challenges for acquiring conditions for starting OH initiatives, their causes and definitions. Policy prioritization and funding, and educational and training programs are essential conditions for initiating OH initiatives. The reference numbers follow the notation presented in the Reference list.

Conditions for starting	
**Policy and funding** [5,[17], [18], [19], [20], [21], [22], [23], [24], [25], [26], [27], [28], [29], [30], [31], [32], [33], [34], [35], [36], [37], [38], [39], [40], [41], [42], [43], [44], [45], [46]]	**Causes and defining arguments**
Lack of resources and funding for OH initiatives [[18], [19], [20],^22^,^23^,^30^,^31^,^37^,^39^,^44^,^45^]	• OH initiatives need unified and large-scale funding, which is not only hard to acquire but also difficult to coordinate, which lead to the preference for funding disease-specific programs [19,37,44] • OH initiatives have to compete for scarce resources with (single) disciplinary and specialized projects [19,22,37,39] • Governmental funders prioritize security and economy over health and epidemic preparedness [18,30,31,45] • Donors prescribed research agendas generate fragmentation in resource allocation [20,22,44] • Donors do not coordinate between themselves neither engage with local governments and health systems, generating inefficient resource allocation [18,23]
Lack of overall awareness about OH [5,17,19,21,22,25,26,[28], [29], [30], [31], [32],[37], [38], [39], [40],^44^]	• Inefficient and insufficient advocacy and message development from OH practitioners [5,17,19,25,26,[28], [29], [30], [31],^38^,^40^]
Lack of commitment of policy-makers with OH [18,[21], [22], [23], [24],^30^,^31^,^45^]	• OH initiatives are not usually related to only one Ministry, and the collaboration between Ministries to agree in a funding strategy is difficult [18,30,31,45] • Priorities of funders shift to single diseases during emergency situations [[21], [22], [23], [24]]
**Education and training** [2,4,[10], [11], [12],^18^,^19^,^21^,^25^,^28^,^32^,^37^,[43], [44], [45], [46], [47], [48], [49], [50], [51], [52]]	**Causes and defining arguments**
Lack of competence from OH practitioners [4,11,21,25,28,43,47,48]	• Lack of OH practitioners trained to facilitate and coordinate collaborations within OH projects [4,11,28,43,47,48]
Insufficient and inefficient OH training programs [2,18,25,32,37,46,[49], [50], [51], [52]]	• Lack of training programs on collaborative approaches (ID/TD) [18,32,37,46,49,52] • Existing programs are not ideal due to the lack of field training, cross-cultural experiences, and development of managerial and interpersonal skills [46,[49], [50], [51]] • Unequal distribution of scholarly resources, with higher presence in developed nations [2,25]
Lack of academic and institutional support for OH [4,10,12,25,32,37,[45], [46], [47],^49^]	• Lack of academic ID/TD structure encouraging collaborative research and projects [4,46] • Lack of publishing networks, and a well-established recognition and rewarding structure for ID/TD research projects [46,47] • Academic preference for disciplinary/specialist training [10,12,32,37,45,49] • OH programs do not fit within rigid curricula and short time-frames [10,12,32,37,45,49]

Policy support, access to funds, and trained professionals, able to understand and implement the OH approach, are basic conditions to facilitate the start and smooth execution of OH initiatives. Therefore, addressing condition challenges is considered an important first step towards the successful design and implementation of OH initiatives [2]. Some authors also elaborate on the interdependency of these themes [26,29,[44], [45], [46],^53^]. While on the one hand, without policy support and funding, educational and training programs cannot be enhanced; on the other hand, educated and trained personnel are necessary for improving and increasing OH advocacy and message development for policy-makers and the public, and therefore enhancing policy support and funding.

#### 3.1.2 Execution

For those initiatives that are able to meet the necessary conditions for starting, many practitioners also encounter challenges on the next step of their execution (see Table 2). Next to the difficulties in performing OH surveillance, execution challenges relate to problems in collaboration between multiple actors, in multiple domains and at multiple levels. Most of these challenges are perceived at the local (project) level, while performing the experimental initiatives. Nevertheless, the roots of such challenges usually come from structures institutionalized at a systemic level.

**Table 2:** Challenges for executing OH initiatives, their cases and definitions. Difficulties in performing OH surveillance can be seen as an example of challenges that occur while executing OH initiatives, as well as problems endured during collaboration. The reference numbers follow the notation presented in the Reference list.

Execution	
**Surveillance** [2,5,9,19,[22], [23], [24],[29], [30], [31],^35^,^36^,^40^,^41^,^43^,^45^,^46^,^49^,[54], [55], [56], [57]]	**Causes and defining arguments**
Hard to perform OH surveillance [19,22,31,41,45]	• Logistical challenges such as lack of resources and personnel [19,22,31,41] • Lack of legal basis for integrated surveillance across different domains (environmental, animal and human health systems) [45]
Problems with access to and quality of OH data and information [24,29,30,35,36,40,45,49,[54], [55], [56]]	• Missed/delayed identification of causes of diseases due to lack of access to integrated/combined data [36,56] • Lack of/delay sharing of epidemiological and molecular data [24,29,30,35,36,45,54,56] • Insufficient/inefficient data collection in some local/national surveillance systems [54] • Lack of data standards (for date collection and analysis) [24,29,36,54,55] • Lack of data quality [24,56] • Extended effort/time needed to standardize and integrate data [24,36,49,54,55] • Bad reporting systems and lack of databases/sharing platforms [24,40,49,55]
Lack of surveillance capacity [2,5,9,19,[22], [23], [24],^30^,^31^,^36^,^45^,^46^,^55^,^57^]	• Uneven geographic distribution of surveillance capacity [5,9,22,30,57] • Lack of laboratory/infrastructure capacity [9,23,24,30,31,46,55] • Lack of human resources to perform surveillance [2,9,19,30,31,36] • Weak health systems defaulting health service delivery [2,9,23,31,46] • Unsustainable (donor) funding [23,46]
Fragmented surveillance systems [9,19,23,30,40,43,45,54,55]	• Hard to integrate animal, environmental and public health surveillance systems [30,40,54,55] • Lack of surveillance networks [9,40,43] • Poor coordination of surveillance activities [9,19,23,45,55]
**Multi-actor collaborations** [2,4,5,[9], [10], [11], [12],[17], [18], [19],^21^,^22^,[25], [26], [27],^29^,^31^,^34^,^35^,^37^,[41], [42], [43], [44], [45],^47^,^53^,^55^,^56^,[58], [59], [60], [61], [62], [63], [64]]	**Causes and defining arguments**
Difficulties to promote and sustain OH collaborations [10,17,21,22,27,29,34,35,41,42,45,47,56,[58], [59], [60],^62^,^64^]	• Establishing OH collaborations and trust can be complex and time consuming [29,34,47] • Hard to sustain the engagement of stakeholders within OH teams [35,58]
Unequal power/representation of actors [2,9,10,12,18,19,21,22,26,27,31,35,37,42,45,55,56,58,59,[61], [62], [63]]	• Underrepresentation of environmental scientists [2,10,19,21,27,35,37,42,45,59,61,63] • Underrepresentation of social scientists [10,19,26,27,35,56,59,61] • Underrepresentation of economic scientists [19,27,35,56,59] • Underrepresentation of anthropological scientists [62] • Underrepresentation of wildlife scientists [26] • Underrepresentation of human health scientists [31,45,55] • Competition between OH actors [9] • OH is seen as a veterinary-driven initiative [12,18,22,31,35,45]
Lack of facilitated collaborative process [9,10,17,18,21,27,29,35,42,43,45,56,59,60,62]	• Lack of coordination of collaborations [9,18] • Lack of planning of collaborations in different phases (e.g. design, execution) [43] • OH practitioners fail to involve actors outside academia such as community actors [10,21,29,35,60] • OH practitioners fail to address the context-specific issues such as health systems' needs and the interests of local actors [17,27,42,45,56,59,62]
Disciplinary and cultural silo thinking [4,[10], [11], [12],^18^,^19^,^31^,^34^,^35^,^37^,^41^,^44^,^46^,^47^,^58^]	• Hard to collaborate outside one's own epistemic culture [4,11,37,47] • Stakeholders have different perceptions/priorities/interests [10,12,18,31,34,35,41,44,47,58] • Hard to integrate different frameworks/concepts/methods/languages [4,35,47] • Diverse cultural backgrounds [19,34]
**Multi-domain collaborations** [4,10,17,20,22,27,[32], [33], [34],^37^,[39], [40], [41], [42],[44], [45], [46], [47], [48], [49],^52^,^55^,[57], [58], [59],^61^,^65^]	**Causes and defining arguments**
Lack of facilitated collaborative process [4,25,[32], [33], [34],^37^,^40^,^44^,^45^,^48^,^49^,^55^]	• Lack of leadership in OH teams [4,32,44,48] • Lack of trained personnel [25,32,37,40,44,48,49] • Lack of efficient communication within OH teams [33,34,45,55]
Difficulties in promoting the engagement of multiple actors across domains [4,10,17,20,22,33,39,[44], [45], [46], [47], [48],^52^,^53^,^57^,^58^,^61^,^65^]	• Political fragmentation [45] • Hard to incorporate input from multiple actors in research design and analysis [20,47,52,58] • Hard to build trust between stakeholders [33] • Hard to find consensus due to multiple agendas [33] • The engagement of multiple actors can be time consuming [4,10,33,44] • Hard to engage the private sector [39,61] • Competition between stakeholders [57] • Different backgrounds/power/languages/knowledge among relevant stakeholders [4,17,22,33,48,57] • It is hard to integrate diverse perspectives and at the same time respect differences [53,65]
Difficulties to include context-specific factors in OH initiatives [17,27,33,39,41,44,58,59]	• Hard to promote community engagement [17,41,44] • Hard to find tailored solutions and promote changes [27,33,39,58,59] • Hard to consider contextual factors in all its complexity in OH projects [33]
**Multi-level collaborations** [2,17,21,22,25,37,45,46,48,52,58,64,65]	**Causes and defining arguments**
Institutional and academic fragmentation [17,21,22,25,37,46,57,58]	• The integration of high-level health management strategies generate extra costs [21] • Bureaucracy and administrative hurdles as complex web of mandates and jurisdictions make integration difficult [60] • Different organizational structures as administrative locations, availability of personnel and resources [17,25,37,46,57] • Lack of a coordinating body able to promote collaboration and integration of structures and strategies [22]
Geographic and cultural fragmentation [2,22,48,52]	• Territorial and nationalistic behaviour [2,22,48,52] • Global differences especially in cultural practices and disparities in terms of capacity [2,22,48,52]

The challenges affecting the execution of OH initiatives are highly influenced by problems in acquiring conditions, which already started in the initial phase and persist until the monitoring and evaluation. This is especially the case for the difficulties in performing OH surveillance, which are mainly caused by the poor availability of resources and personnel [2,9,19,30,31,36]. The challenges in stakeholder collaboration relate to the fact that a multidisciplinary team of scientists, working together but within their own silo, is not enough for the knowledge co-creation proposed in OH innovations. Nevertheless, such superficial collaborations are a long-term heritage of fragmented systems and practices that influence the flexibility of actors and their organizations to collaborate and integrate diverse ideas, methods and actions [2,22,48,52].

#### 3.1.3 Monitoring & evaluation

To facilitate the adoption, upscaling and institutionalization of OH initiatives, clear evidence of the added value of performing such initiatives needs to be provided. However, a range of challenges relate specifically to the difficulties in performing monitoring and evaluation of OH initiatives (see Table 3).

**Table 3:** Challenges for monitoring and evaluating OH initiatives, their cases and definitions. In order to prove the benefits of performing OH initiatives, OH monitoring and evaluation needs to be improved, by tackling challenges in performing evaluation studies and developing specific OH indicators and metrics. The reference numbers follow the notation presented in the Reference list.

Monitoring & evaluation	
Evidence [2,11,[17], [18], [19],^22^,^24^,^29^,^35^,^38^,^39^,^42^,^45^,^47^,^52^,^54^,^58^,^61^,^64^]	Causes and defining arguments
Lack of OH evaluation studies and reporting of outcomes [11,19,22,24,38,42,45,52,54,61]	• Lack of cost-effectiveness analysis for OH initiatives [11,38,42] • OH initiatives require long term monitoring [52] • OH is a relatively recent approach and evaluation studies are not yet completed [24,52]
Lack of guidelines and metrics for OH monitoring and evaluation [17,24,29,35,38,42,47,58]	• Lack of qualitative and quantitative indicators for OH outcomes' measurements [17,58]

Poor monitoring and evaluation of OH initiatives hamper performance assessment of prior (implemented) initiatives and therefore the gathering of evidence on their effectivity and efficiency. Challenges in monitoring and evaluating OH initiatives were mentioned as major, and it was argued that they hinder more widespread political interest and support for the OH approach [24,52].

### 3.2 Causal relation of challenges

**Fig. 2:**
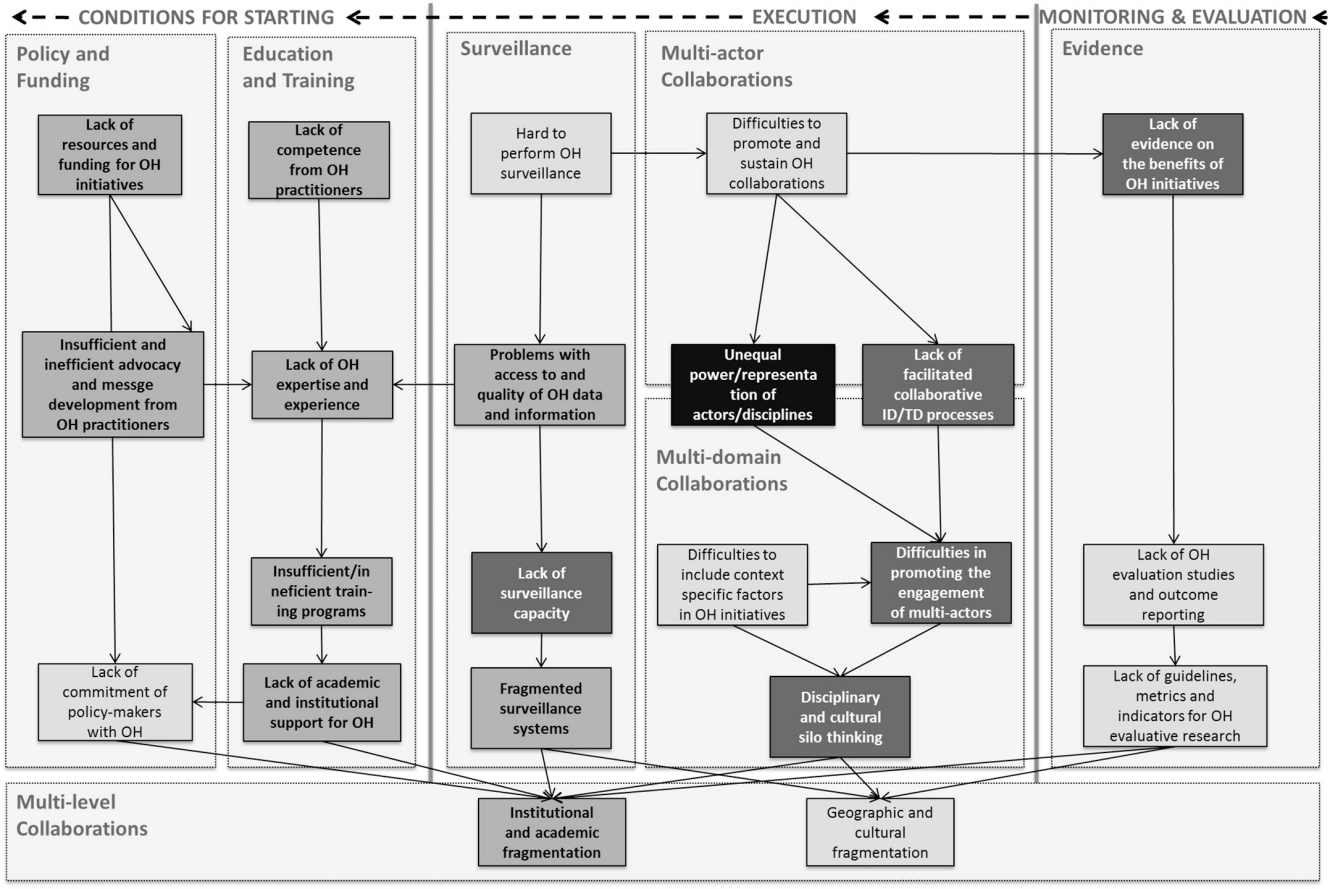
Simplified causal tree. This simplified version of the causal tree depicts groups of challenges showing that although challenges were organized in different process phases and themes, they are interconnected through overlapping causes, crosscutting causal relations and even direct links. The colour code scheme represents the frequency in which the included papers mentioned the challenges, with darker colours representing highly mentioned and lighter colours representing less frequently mentioned.

**Fig. A.1:**
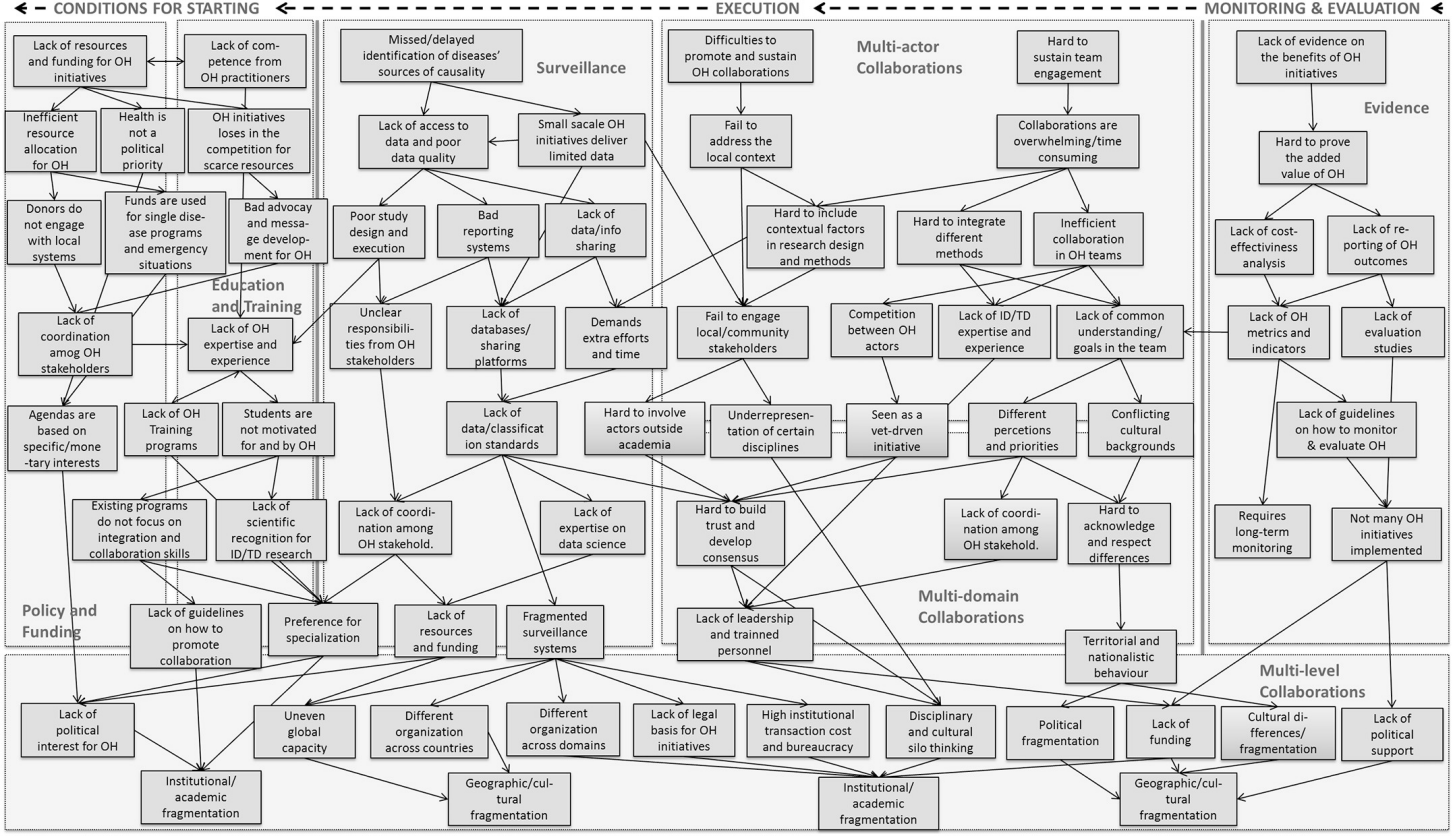
Causal tree of challenges. The causal relations between challenges for performing OH initiatives are depicted through the representation of the logical structure of causal arguments [15]. Challenges are broadly classified in process phases (conditions for starting, execution and monitoring and evaluation), and grouped in themes (policy and funding, education and training, surveillance, multi-domain collaborations, multi-actor collaboration, multi-level collaborations, and evidence).

After the identification of individual challenges, a causal analysis was performed to unravel the causal relations between the different challenges, through the construction of a causal tree. For an overview of the complete causal tree of challenges, refer to Fig. A.1 in the Appendix. The tree follows a line of argumentation in which causal factors for challenges are hierarchically clustered. As a result causes that are (perceived as) more tangible are at the top and causes that are more fundamental and embedded in the system are at the bottom [66]. In Fig. 2, a simplified version of the causal tree is presented depicting the interconnection between the main (groups of) challenges and positioning them according to the previously described causal line of argumentation. The root-causes for all types of challenges are placed at two different types of fragmentations. The institutional-academic fragmentation relates to differences in educational and professional pathways, and organizational structure, resulting in diverse methodological preferences, practices, background assumptions and normative orientations of actors [[67], [68], [69]]. The geographic-cultural fragmentation is embedded in historical, geographic and social factors, resulting in conflicting actions, values and even disparities in knowledge and capacity.

Added to the qualitative analysis, a semi-quantitative assessment of the frequency in which the different challenges were mentioned by the included papers was performed. Most of the included papers mentioned challenges located at the phase of execution, followed by challenges in the conditions for starting and finally challenges for the monitoring and evaluation phases. Regarding the thematic classification, most papers mentioned challenges related to multi-actor collaborations (*n* = 36); followed by policy and funding (*n* = 31), and multi-domain collaborations (*n* = 27), respectively. Equally mentioned were the themes of surveillance, and education and training (*n* = 22). The least mentioned themes were evidence (*n* = 19) and multi-level collaborations (*n* = 13).

### 3.3 Defining solutions for OH challenges

Next, the 56 included papers were analysed for solutions addressing the challenges in designing and implementing OH initiatives. Following the same thematic analysis used to classify challenges, solutions were organized according to phases and themes. In addition, solutions were discriminated on their status of implementation by indicating whether they were only recommended by the authors or also implemented in practice. The implemented solutions serve as examples of strategies that attempted to solve challenges for performing OH initiatives in practice. Notably, the papers not always described solutions for the exact same challenges mentioned in their content. Some articles proposed solutions for only few of the mentioned challenges, while others presented solutions beyond mentioned challenges. For this reason, solutions are not linked to individual challenges but rather broadly related to the thematic groups.

#### 3.3.1 Solutions for improving conditions for starting

The solutions proposed for boosting the start of OH initiatives aim at acquiring and establishing the necessary conditions such as improving political support and access to funds, and enhancing the educational and training opportunities for OH practitioners (see Table 4).

**Table 4:** Solutions proposed for improving conditions for starting OH initiatives. In order to start and further execute OH initiatives, policy support and access to funds need to be improved, as well as the amount and quality of OH educational and training programs. The reference numbers follow the notation presented in the Reference list.

Improving conditions for starting		Status of implementation
1. Policy and funding [2,4,9,18,19,[21], [22], [23],^26^,^27^,^29^,^31^,^32^,^34^,[37], [38], [39], [40], [41],[44], [45], [46],^48^,^49^,^52^,^53^,^55^,^58^,^64^]	1.a OH practitioners should work together with international organizations, other governments and NGOs to leverage funding and support for OH initiatives [18,19,21,22,27,31,34,39,45,46,49,52,55,58,64]	Implemented [18,19,21,22,31,34,39,45,46,49,52,55,58,64]
	1.b Increase policy-makers awareness about the struggles to implement OH initiatives (timelines and need for long-term support) [32,46]	Recommended
	1.c Increase public awareness about OH and its benefits [2,9,26,34,37,49]	Implemented [49]
	1.d Involve policy-makers in the design and execution of OH initiatives [26,38,39,41,48,52,58]	Implemented [41,48,52,58]
	1.e Improve OH advocacy [23,32,39,41,49,53]: • By using a team approach for developing message, requesting and using funds [26,29,[44], [45], [46],^53^] • By understanding and influencing policy (theory of change) [18,19,22,31]	Implemented [23,32,39,41,44,46,49,53]
	1.f Create a unified funding structure that can be used by OH initiatives and their practitioners to improve the health of local communities [4,9,21,31,40,41,45]	Implemented [4,9,21,31,40,41,45]
	1.g Adopt a more grounded national perspective, by building OH initiatives with local funding and in local infrastructure [4,18,21,23,31]	Implemented [4,18,21,31]
	1.h Develop adequate evidence-based policies [18,21,31,40,46,52,53]	Implemented [21,31,52]
2. Education and training [2,4,10,18,19,21,22,27,28,31,32,34,37,40,41,[43], [44], [45], [46],[48], [49], [50], [51], [52],^58^]	2.a Develop online OH educational programs especially for developing countries [2,46]	Implemented [46]
	2.b Procure funds for training opportunities from international organizations [18,21,34,37,40,50,52]	Implemented [18,21,34,37,40,50,52]
	2.c Implement field-based training with community engagement [4,10,21,32,41,44,[48], [49], [50],^52^,^58^]	Implemented [21,32,41,44,49,58]
	2.d Offer training in TD methods (e.g. participatory, community-based methods, system thinking theory) [4,10,21,32,48,50,52]	Implemented [50,52]
	2.e Offer training in interpersonal skills (leadership, human resources management, health diplomacy, communication) [4,27,28,32,45,[48], [49], [50]]	Implemented [45,[48], [49], [50]]
	2.f Offer training in epidemiology and environmental sciences [10,21]	Implemented [21]
	2.g Offer training in social sciences (sociology, anthropology, economics) [10,50]	Implemented [50]
	2.h Establish optional and supplemental OH study programs [19,22,37,40,43,45,51]	Implemented [19,22,40,43,45,51]
	2.i Improve networking of study programs with different countries to expose students to cultural differences [34,[48], [49], [50],^52^]	Implemented [34,49,50,52]
	2.j Implement truly ID research, educational departments and degree programs [22,32,51,52]	Implemented [52]
	2.k Establish training for future OH practitioners in partly overlapping curricula [45,49]	Implemented [45,49]
	2.l Implement OH training programs at different levels (academia, government, NGOs and community) [4,10,18,19,22,40,46,49,52]	Implemented [18,19,22,40,49,52]
	2.m Restructure how researchers are evaluated and rewarded within academic institutions [4,32]	Recommended

The solutions proposed to improve policy support and funding for OH initiatives elaborate especially on enhancing OH advocacy through better message development and collaborations with policy-makers. The solutions proposed to improve OH education and training emphasize the need of implementing ID and TD training programs and educational departments, as well as taking a more holistic approach to educational programs with the inclusion of different disciplines and courses on interpersonal skills. For only two solutions no implemented examples were mentioned: 1) strategies to increase policy-makers awareness about the struggles to implement OH initiatives (1b); and 2) the need to restructure how researchers are evaluated and rewarded within academic institutions (2 m). Although the first solution seems easy to implement, meaningful results would only be generated if there is a change in behaviour, in which policy makers increase their support for OH. The second solution is clearly a challenging suggestion, since that would imply changing the way the academic and scientific community is structured for many years, which can face a lot of resistance from the stakeholders involved.

#### 3.3.2 Solutions for improving execution

The solutions proposed for the execution challenges focus on the following themes: improving OH (integrated) surveillance; and enhancing multi-actor, multi-domain, and multi-level collaborations (see Table 5).

**Table 5:** Solutions proposed for improving the execution of OH initiatives. In order to improve the execution of OH initiatives, solutions should focus on enhancing OH surveillance, and collaborative efforts between the multiple actors, among multiple domains, and at multiple levels. The reference numbers follow the notation presented in the Reference list.

Improving execution		Status of implementation
3. Surveillance [2,9,17,19,[21], [22], [23], [24],^30^,^35^,^36^,^39^,^40^,^45^,^48^,^49^,^51^,^54^,^55^,^57^,^61^,^63^]	3.a Consider endemic diseases (not only epidemics) for OH surveillance [2,23,24,45]	Implemented [2,23,45]
	3.b Use (public and private) diagnostic laboratories as a source for obtaining surveillance data [45,54]	Implemented [45,54]
	3.c Use molecular data in OH surveillance systems [35]	Implemented [35]
	3.d Use alternative data sources (online data from social media and local news reports) to leverage big data [24,36,40,61,63]	Implemented [24,36,40,61,63]
	3.e Improve data reporting systems [30,35,48,54,55,61]	Implemented [30,35,54,55,61]
	3.f Develop information flow systems (networks, databases, sharing platforms) [2,9,22,24,30,36,39,40,45,49,51,54,55,57]	Implemented [9,22,24,30,36,39,45,49,51,55,57]
	3.g Implement sharing systems directly with the data providers [54]	Implemented [54]
	3.h Develop data standards [54,57]	Implemented [57]
	3.i Develop guidelines and agreements for data sharing [9,17,45,48]	Implemented [9]
	3.j Improve laboratory and operational capacities for OH surveillance [2,40,55]	Recommended
	3.k Implement permanent multi-sectoral (OH) rapid response teams [23,55]	Implemented [23,55]
	3.l Acknowledge and implement mechanisms to assure a legal basis for joint surveillance activities among countries [45,48,49,55]	Implemented [48,49,55]
	3.m Implement sustained capacity building of OH personnel [19,21,40,49]	Implemented [49]
4. Multi-actor collaborations [4,9,10,[17], [18], [19],[21], [22], [23],[25], [26], [27],^29^,^30^,^32^,^34^,^37^,[39], [40], [41],[43], [44], [45], [46], [47], [48], [49],[51], [52], [53],^55^,^56^,[58], [59], [60], [61], [62], [63], [64]]	4.a Take a more holistic approach to OH collaborations and analysis methods by including different disciplines/actors [19,22,29,40,41,48,49,53,58] • Include all foundational disciplines/processes underlying health ecologies [9,10,25,56,59,62,64] • Improve the inclusion of actors/disciplines from social and environmental fields [9,10,27,45,56,[60], [61], [62], [63]]	Implemented [10,19,22,29,40,41,45,48,49,53,56,58,60,[62], [63], [64]]
	4.b Apply participatory methods (involve community members) [4,10,[17], [18], [19],[25], [26], [27],^32^,^40^,^44^,^45^,^48^,^52^,^53^,^58^,^60^,^62^]	Implemented [4,19,32,40,41,44,48,52,53,58,60]
	4.c Improve communication across disciplines (e.g. through ID conferences, developing a common language, active listening, political finesse) [4,9,17,19,22,23,27,37,39,41,45,46,49,55]	Implemented [4,9,17,19,22,23,37,39,41,45,46,49,55]
	4.d Focus on building the project around strengths and opportunities by considering the comparative advantages of each expert/discipline [18,41]	Implemented [41]
	4.e Need to reduce conflict and perceived power differentials among disciplines [4,32,34]	Implemented [34]
	4.f Ensure team members are not only diverse (multidisciplinary approach) but also interact and work together in knowledge co-creation [17,25,34,39,41,45,47,49,61] • Ensure actors avoid silo thinking and are receptive to ideas from actors with different backgrounds [9,58]	Implemented [34,39,41,45,49,58]
	4.g Improve communication and coordination within OH teams [30,39,49,52] • Implement early discussions in a neutral environment for developing common terminology, framework, goals and interests with transparency [4,9,10,18,25,34,41,[45], [46], [47], [48],^58^] • Establish shared project leadership [48]	Implemented [30,34,39,41,49,52]
	4.h Design and coordinate knowledge sharing platforms, methods and protocols [10,17,34,39,45,47,48,51,52]	Implemented [39,45,51,52]
5. Multi-domain collaborations [4,11,21,22,27,28,[32], [33], [34], [35],[39], [40], [41], [42], [43], [44],[46], [47], [48],^52^,^53^,^55^,^56^,[58], [59], [60], [61],^65^]	5.a Build personal relationships among actors based on transparency, trust and respect [11,22,27,35,40,41,44,46,48,52,60] • Improve communication with community actors, with clear message and using the local language [27,28,33,55,58] • Discuss potential unexpected results with the involved stakeholders [33] • Deemphasize differences in status and power between the different stakeholders [4] • Do not compress variation but try to attend differences [65]	Implemented [4,22,28,35,40,41,44,52,60]
	5.b Develop guidelines for TD collaboration, as a TD roadmap that will help teams to delineate leadership roles and responsibilities [4,21,39,43]	Implemented [39]
	5.c Ensure that actors first find consensus within their own domain before collaborating across domains [22]	Recommended
	5.d Engage partners and stakeholders in OH initiatives in an early stage [39,41,48]	Implemented [39,41,48]
	5.e Keep the OH approach flexible, open to new ideas and contributions [22,42,47,53,56,58,59,61]	Implemented [22,42,56,58,61]
	5.f Recognize the long duration of TD/OH research projects [34]	Implemented [34]
	5.g Recognize and reward (incentives) for OH facilitators, team members and community actors [27,28,48,54]	Implemented [28]
	5.h Establish skilled OH leaders and facilitators [4,11,22,27,32,33,48]	Recommended
6. Multi-level collaborations [2,5,9,10,[17], [18], [19],[21], [22], [23],^31^,^32^,^34^,^40^,^41^,^43^,^45^,^48^,^49^,^52^,^53^,^55^,^57^,^58^,^60^,^64^]	6.a Improve collaboration between ministries [18,21,31,43,45,52]	Implemented [18,21,31,45,52]
	6.b Formulate shared visions, regulations, and memoranda of understanding for mainstreaming OH approaches [9,34,43,48,52,55,60]	Implemented [9,34,52,55,60]
	6.c Facilitate international and national efforts to increase inter-sectorial collaboration and coordination at national level, through actions by international organizations (FAO, OIE, WHO, WTO and EU) and associations [9,18,21,22,31,40,43,45,48,57,64]	Implemented [9,18,21,22,31,40,45,48,57]
	6.d Operationalize and sustain practical applications of OH at ground level through institutional innovation [2,[17], [18], [19],^21^,^22^,^48^,^49^,^52^,^64^] • Improve the understanding of institutional missions, capacities, roles and responsibilities [5,34,53] • Improve knowledge of public health organizations regarding complex systems and the recognition of the importance of OH, besides enhancing their capacity and ability to work in an ID/TD context [9,22]	Implemented [2,19,21,34,49,52,53,64]
	6.e Build core coordinating capacity to improve integration, coordination and collaboration at institutional, scientific and geographic level [2,10,17,19,[21], [22], [23],^45^,^48^,^49^,^58^] • Identify individuals to become OH ‘champions’ at a political level [18,31] • Decide on an operational scope of OH, agreed among key global institutions [22]	Implemented [18,22,45,48,49,58]
	6.f Focus on the strengthening of the community model health system for a better sustainability [2,32,40,41,52]	Implemented [32,40,41,52]
	6.g Use top-down governance mechanisms to steward OH initiatives [17]	Recommended

The solutions proposed for improving OH surveillance focus mainly on enhancing the integration and sharing of surveillance data, added to capacity building of infrastructure and human resources. In this group, only for the suggestion of improving laboratory and operational capacities for OH surveillance (3j) no implemented example was identified; indicating that capacity building in OH surveillance, although important, has been extremely challenging. Improvements to the number and quality of OH facilitators, who can promote better collaboration, communication, and coordination between stakeholders, were mentioned as an important solution for all three levels of collaborations. For improving multi-actor collaborations, the identified solutions mainly propose the improvement of superficial collaborations by overcoming silo thinking through the support and facilitation of knowledge co-creation among actors. Solutions for multi-domain collaborations aim at creating engagement and trust among actors from all relevant sectors and domains. Still, two of the proposed solutions in this group appear to be challenging for implementation: 1) the need to ensure that actors first find consensus within their own domain before collaborating across domains (5c); and 2) establishing skilled OH leaders and facilitators (5 h). Similar to the case of surveillance, this last recommended solution highlights the difficulty in implementing strategies focused on capacity building for OH, not only at a structural level, but also in a human capacity level. Specific for multi-level collaborations, solutions were proposed in a much more managerial and governance level, therefore the engagement of national and international policy-makers is perceived as essential [18,22,43,57,64]. One solution in this group that was only recommended was the suggestion to use top-down governance mechanisms to steward OH initiatives (6 g). An explanation for that can be that the most common way of implementing and stewarding health related interventions is through a top-down approach; the opposite approach (bottom-up) might be seen as more innovative and therefore worthy of mentioning.

#### 3.3.3 Solutions for improving monitoring & evaluation

The solutions proposed for improving OH monitoring and evaluation aim at generating reliable evidence on the benefits of performing OH initiatives (see Table 6).

**Table 6:** Solutions proposed for improving the monitoring and evaluation of OH initiatives. In order to improve OH monitoring and evaluation, more studies need to be performed, guidelines developed, and specific OH metrics and indicators proposed. The reference numbers follow the notation presented in the Reference list.

Improving monitoring & evaluation		Status of implementation
7. Evidence [5,17,23,24,29,30,32,38,40,42,44,45,53,[61], [62], [63], [64]]	7.a Develop a standardized framework for systematic evaluation and reporting of OH outcomes [24,30,38]	Implemented [24]
	7.b Establish a network, as a community of experts, to develop science-based evaluation protocols for OH [42]	Recommended
	7.c Perform cost-effectiveness analysis and develop a OH business case [5,32,38,40,42,45]	Implemented [5,32,38,42,45]
	7.d Consider evaluation before program implementation (in the design phase) [23,38]	Implemented [23,38]
	7.e Include measures relevant to each sector in the monitoring and evaluation [38]	Implemented [38]
	7.f Develop standardized quantitative indicators for OH evaluation [24,42]	Implemented [24]
	7.g Use examples from other disciplines for improving monitoring and evaluation (e.g. epidemiology, environmental impact, socio-economics) [29,38,53,61] • Use the outcome mapping technique [30]	Implemented [29,38,53,61]
	7.h Understand health as a “quantitative and qualitative interaction and outcome process in social-ecological systems” [17,29,38,42,44,53,[61], [62], [63]]	Implemented [29,38,42,44,53,61,63]
	7.i Test and monitor the development and use of OH metrics and indicators [29,45]	Implemented [29]

The solutions proposed for improving OH monitoring and evaluation elaborate on different gaps on the existing evidence on OH outcomes: the absence of studies and guidelines for OH monitoring and evaluation, and the need of developing holistic OH metrics and indicators that account for both quantitative and qualitative aspects. Within this group, only one solution was not accompanied by an example of implementation: the establishment of a network of experts to develop evaluation protocols for OH (7b). Although a proof-of-principle for this solution was not provided, it might be an interesting approach to try as an attempt to improve OH monitoring and evaluation.

### 3.4 Solutions at a multi-level perspective

**Fig. A.2:**
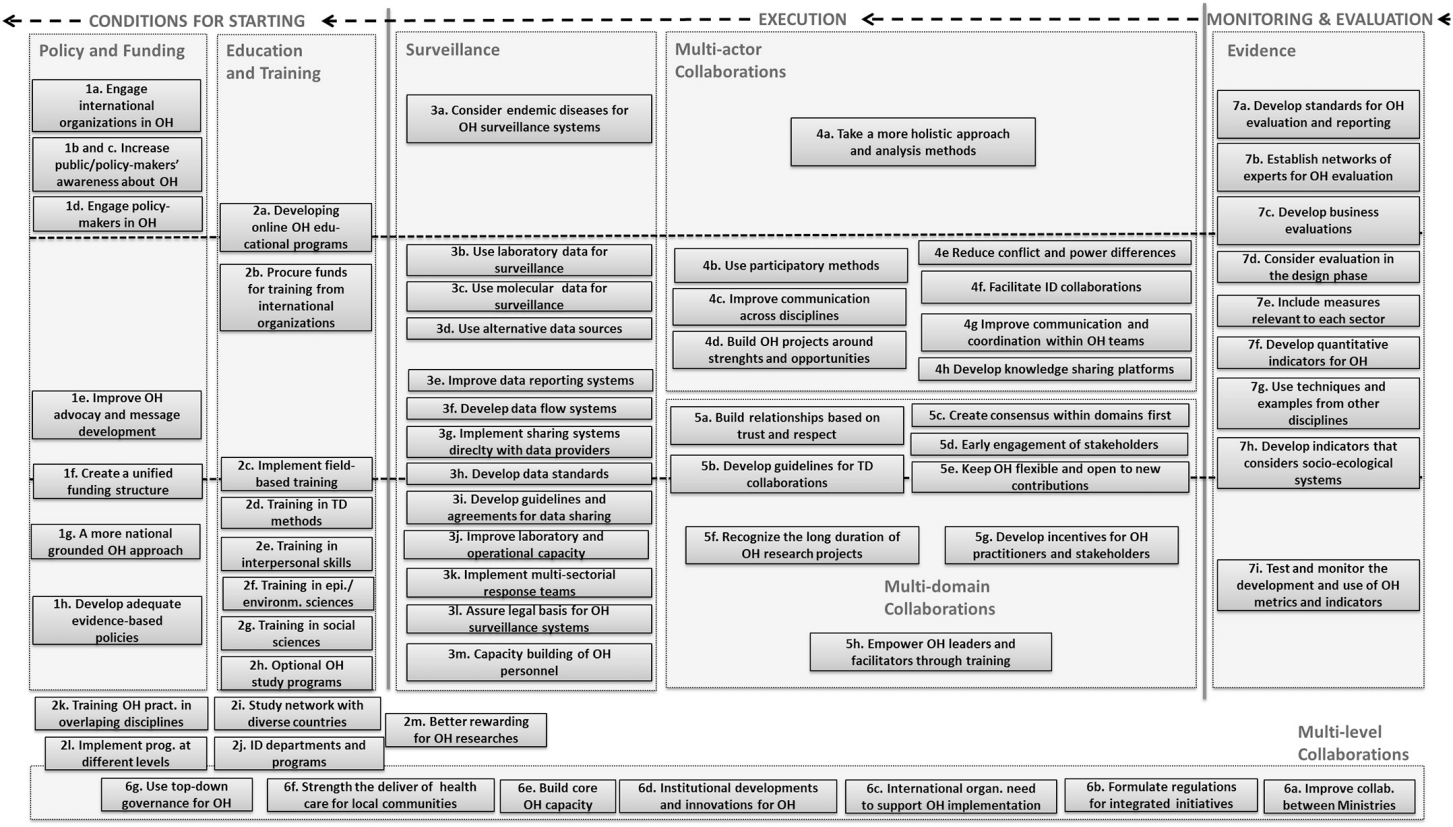
Solutions mapped to the causal tree. The mapping of solutions on the causal tree reveals which challenges specific solutions aim to address (according to Fig. 2, Fig. A.1): symptomatic/superficial challenges, on the top; or fundamental/systemic challenges on the bottom. In this sense, solutions at the middle of the causal tree (in-between dotted lines) have a focus on local and immediate problems, being easier to implement with the potential for generating faster results. Solutions are also mapped in relation to the different process phases and themes.

The solutions were also plotted in the framework of the causal tree of challenges (see Fig. A.2 in the Appendix) in order to visualize how they relate to the causal line of argumentation. In fact, the causal tree (displayed in Fig. 2, Fig. A.1) is essential to understand why and how interventions and solutions may or may not work. Since more tangible challenges are at the top of the tree, solutions that focus on these rows tend to be superficial, targeting symptoms rather than the real causes. On the other hand, solutions focused on the bottom rows of the tree tend to be hard to implement, since they tackle fundamental problems embedded in institutions and systems. It follows that most of the solutions for which no examples of successful implementation (i.e. recommended solutions) were provided, were located either at the very beginning (1b and 7b) or at the very end (2 m, 3j, 5 h and 6 g) of the tree. Tackling causes in the middle of the causal tree tends to be easier and therefore more implemented examples were provided. In addition, adopting solutions that address a cause for multiple symptomatic challenges is also beneficial, since it can solve several challenges at once, having the potential to generate faster results [66]. Furthermore, even if the papers mentioned solutions that were successfully implemented, those usually occurred on a small-scale and in specific contexts. Moreover, although a solution worked in a specific context, it did not solve all the challenges that the specific OH initiative was facing; most papers still described enduring challenges even after the solutions were implemented. These enduring challenges were usually related to a different theme, and sometimes even phase, from the one that the implemented solution addressed. This confirms the interdependency of challenges, which indicates that in order to successfully implement OH initiatives, a range of solutions need to be considered to tackle simultaneously the different themes pertaining to the different process phases.

The frequency in which the included papers mentioned specific solutions was also assessed. The majority of papers propose solutions at the phase of execution, followed by the conditions for starting and finally the monitoring and evaluation phase. In relation to the thematic classification of challenges, most papers mentioned solutions related to the theme of multi-actor collaborations (*n* = 39), followed policy and funding (*n* = 29). Closely mentioned were the themes of multi-domain collaborations (*n* = 28) and multi-level collaborations (*n* = 26). The next most mentioned themes were education and training (*n* = 25) followed by surveillance (*n* = 22). Solutions for the theme of evidence were least mentioned (*n* = 17).

## 4 Discussion

In this paper, we showed that a variety of challenges endured by OH initiatives affect their performance in different process phases and across different themes. The causal analysis revealed that challenges are interconnected through overlapping causes, crosscutting causal relations and even direct links, emphasizing the need for integrative approaches as the OH. The striking majority of mentioned challenges related to problems in the collaboration between the different stakeholders. The root causes for all types of challenges are at the institutional and systemic level, and they relate to two different fragmentations: either the institutional-academic (e.g. professional and organizational silos), or the geographic-cultural (e.g. diverse preferences, values and capacity). Still, many solutions are proposed for tackling the challenges in performing OH initiatives and, for most of these solutions, practical examples of implementation were described. However, even for the challenges of which examples of implemented solutions were described, the initiatives still faced persistent challenges to be addressed in the different phases and themes. The biggest knowledge gap, in terms of proposed solutions for overcoming the challenges endured by OH initiatives, was notably for performing monitoring and evaluation.

A key finding from the causal analysis is that challenges and their causes are interrelated across phases and themes. While the lack of conditions (policy and funding, and education and training) causes difficulties in the execution of OH initiatives, a poor execution is a cause for the difficulties in producing OH evidence; as monitoring and evaluation is better performed when planned already during the design and execution phases. Furthermore, the lack of evidence on the benefits of performing OH initiatives is a cause for the lack of conditions, since to guarantee policy support and funding, evidence about the benefits of performing the OH approach needs to be provided.

In addition, the process phases for performing OH initiatives, hereby delineated, do not happen in a demarcated and unrelated fashion, but rather in an interactive and iterative way. Creating the necessary conditions, for instance, is important not only for boosting the start of initiatives but should be a constant effort in place throughout the whole process and at different levels. For instance, relevant competences by practitioners, and methodological principles at project level are essential conditions for starting the project; still, conditions in the institutional context (organizational flexibility) and correlations with the wider societal context (community engagement and consideration of local context) are essential for generating intended changes. Therefore, efforts have to be made simultaneously to create conditions at project level, and to achieve embedding and support at institutional level [16]. Although this may be true, this paper showed that projects are still initiated and executed even when conditions are not completely present [16]. In fact, even without conducive institutional and cultural conditions at the system level, experiments and initiatives happen. However, under this lack of proper conditions, practitioners struggle with enduring barriers for the implementation and scaling-up of such projects, as demonstrated in this paper for OH initiatives.

This unclear delineation of challenges across categories also applies to their classification in themes. Albeit the thematic classification of challenges has been broadly used by different fields of integrative approaches [68,[70], [71], [72], [73], [74]], it does not reflect how challenges work in practice. This finding also affects the strategic implementation of solutions. Although many examples of implemented solutions were identified, they did not address all problems faced by the described OH initiatives, most authors reflected in their papers on enduring problems on the overall performance of the OH approach. To improve the performance of OH initiatives, a range of solutions should be implemented to tackle simultaneously challenges at the different phases and from different themes. The surveillance of zoonotic diseases, for instance, could benefit significantly from the OH approach, through the integration of data and analysis from human, veterinary and environmental domains. Nevertheless, difficulties in collaboration between these actors lead to inefficiencies in data sharing, integration and collective analysis [30,40,54,55]. Therefore, solutions should focus at the same time on improving the standardization and integration of surveillance data through the enhanced collaboration of relevant stakeholders across domains. Other authors similarly claimed that neglecting the interconnection between different thematic aspects (such as epistemic, regulatory, and practical aspects) can generate inefficiencies in the execution of projects [69].

Not surprisingly, among the manifold challenges endured by OH initiatives that persist in different phases and relate to different themes, this paper shows that challenges for promoting collaboration between the wide diversity of stakeholders, as a fundamental aspect in the OH approach, are by far the most mentioned in the literature. Collaboration challenges are also recognized in collaborative approaches from other ID and TD fields as transition management [[70], [71], [72]], and resilience thinking [[75], [76], [77]]. The emergence of the OH approach challenged the pre-conception that global health problems could be solved through insights from experts working exclusively within their own discipline, proposing a multidisciplinary engagement of stakeholders [69]. However, as showed in the collaboration challenges, the difficulties in promoting real and equal participation from diverse actors persists. The intended innovation proposed by the OH approach, necessary to solve global health challenges, will only occur when stakeholders manage to overcome their professional and cultural silos to work together and therefore generate the co-creation of new knowledge, methodologies and practices [17,25,47,61]. Multi-level collaborations are also problematic, since they request the engagement of policy-makers and international organizations, who tend to interpret the scope of OH within the context of their mandate and activities. Their reluctance to broaden their vision and scope affects the wider acceptance and implementation of collaborative approaches as OH. In this context, some authors argue that this conservative attitude at a preeminent level influences the performance of mainstream OH initiatives, because practitioners tend to reproduce the narrowed pursue of specific goals with the prioritization of self-interests, leading to reductionism and fragmentation [22].

Notably, the root causes not only for collaboration, but all types of challenges are embedded in two different types of institutional and systemic fragmentations. Firstly, institutional-academic fragmentation is reflected on disciplinary and organizational silos, with each institution having specific working practices and methods. Secondly, the geographic-cultural fragmentation is reflected in diverse social and cultural preferences, values and even disparities in knowledge and capacity; which is particularly, but not exclusively, evident in institutions from developed nations adjacent to developing countries. Building upon similar root causes found in the fields of OH data sharing [78] and rabies innovation [14], this study elaborates on fundamental challenges that generate persistent and complex problems for OH initiatives.

Recognizing that the search strategy for including papers in this review focused on identifying challenges for designing and implementing OH initiatives, this review does not provide an exhaustive overview of successfully implemented OH initiatives. Still, the striking majority of the included papers either proposed or discussed solutions to address challenges, and even described examples implemented in practice. Interestingly, most of the solutions for which no example of implementation was found were located either at the beginning or the end of the causal tree. This reflects the logic of the fundamentality of the different challenges and their causes, solutions at the middle level have a focus on local and immediate problems, being easier to implement with the potential for generating faster results. Still, several solutions were implemented at the bottom level. Solutions proposed for the bottom (systemic) level aim at the root causes of the OH challenges, having the potential to generate long-term and sustainable results. Nevertheless, individual OH practitioners can do little in influencing the adoption and implementation of solutions at this institutional and systemic level. Hence, the organization of OH practitioners (e.g. in projects or networks) and engagement of policy-makers, international organizations, and community members are essential preconditions for supporting solutions that propose deeper institutional and cultural changes.

Finally, a mismatch was noticed in terms of most mentioned challenges and proposed solutions. This is especially the case for the challenges under the themes of multi-level collaborations, and evidence. Few papers mentioned specific challenges for multi-level collaborations, however, several papers proposed solutions to address these challenges. As aforementioned, a possible reason for that is the preference for solutions that can cause deeper and sustained changes (at a systemic level), even if they are hard to implement. The opposite happened for the challenges in monitoring and evaluating OH initiatives, where the biggest gap in the literature in terms of proposed solutions was identified. The adoption of the OH approach is unlikely to increase unless a clear sign of its benefits is provided. Therefore, solving these challenges represents the “unfinished agenda” of OH, in the sense that future studies need to be performed aiming to develop a framework for better monitoring and evaluating OH initiatives.

## 5 Conclusion

This paper indicates that many challenges exist and persist for designing and implementing OH initiatives. Although many solutions have been proposed, they were mostly implemented in a small scale and within a specific context. The success of the OH approach does not rely exclusively upon efforts within local initiatives, but also upon changes in cultural, social and institutional practices, at an institutionalized and systemic level [[79], [80], [81]]. In addition, in order to generate a paradigm shift for solving global health problems, a merely multidisciplinary team of experts is not sufficient. Stakeholders should work at an ID and TD level through the integration of academic and ‘real world’ expertise for knowledge co-creation to address OH challenges in an innovative way. Based on the acknowledgement of possible challenges endured by the OH approach and proposed solutions for these challenges, OH practitioners are able to plan and structure the designing and implementation of OH initiatives in a more successful way, by avoiding barriers and/or through the strategic pre-consideration of solutions. Nevertheless, a knowledge gap was identified for solutions proposed to solve challenges in monitoring and evaluating OH initiatives. Future research should focus on this theme in order to provide clear evidence on benefits of using the OH approach.

## Declaration of interest

The authors declare no conflict of interest.

## Funding source

This research did not receive any specific grant from funding agencies in the public, commercial, or not-for-profit sectors.

## Authors' contribution

CdSR performed the data collection and wrote the first draft of the manuscript. LHMvdB and BJR critically revised and edited the manuscript. All authors were involved in conceptualizing and designing the study; analysis and interpretation of data; and final approval of the manuscript.

## Figures and Tables

**Table A.1:** Detailed description of the included papers. An overview of the included papers, describing their authors and reference number, study description and aim, field of the study, country where the study was performed, and the OH definition used.

Authors	Description & Aim	OH Definition	Geography	Field
[32] Allen-Scott, Buntain, Hatfield, Meisser & Thomas	The authors outline key concepts and methods for building individual and institutional TD capacity in order to highlight three value propositions that support improved academic institution capacity for TD OH research.	OH in the TD sense, beyond academic actors	Non-geographically located	The OH approach in itself
[11] Anholt, Stephen & Copes	The authors examine how to build relationships to effective collaborative response to a health problem that crosses disciplinary boundaries. The aim was to support the development of recommendations for effective ID collaboration.	OH in the ID sense, with the integration of veterinary, medical and environmental sciences	Canada	Emerging zoonotic diseases
[33] Anticona, Coe, Bergdahl & San Sebastian	This paper illustrates how knowledge generated from participatory research does not necessarily imply solving a public health problem. This study aimed to contribute to the understanding of the benefits and barriers of following the basic principles of the Ecohealth approach.	Ecohealth in the TD sense, beyond academic actors	Peruvian Amazon	Heavy metal exposure
[12] Atlas	This article discusses about the future of OH. There is a need to more clearly define its boundaries and demonstrate its benefits. Interestingly, the greatest acceptance of OH is seen in the developing world where it is having significant impacts on control of infectious diseases.	OH in the ID sense, with the integration of veterinary, medical and environmental sciences	Non-geographically located	The OH approach in itself
[36] Asokan & Asokan	The major obstacles to control zoonotic diseases include insensitive systems and unreliable data. This article argues that intelligent handling big data can accomplish the overall goals of OH.	OH in the ID sense, with the integration of veterinary, medical and environmental sciences	Non-geographically located	Zoonosis
[63] Barrett & Bouley	The authors assessed the current integration of environmental issues in OH publications and leadership, to understand its maturation; they gauge environmental representation in OH; and suggest ways to enhance integration of environmental drivers, data, and disciplines into the future development of OH.	OH in the ID sense and advocate for the adoption of the Ecohealth approach in the TD sense	Non-geographically located	The OH approach in itself
[37] Barrett, Bouley, Stoertz & Stoertz	The aim of the study is to offer a vision for improving tertiary education to prepare environmental and health professionals to address a changing world.	OH in the ID sense, with the integration of veterinary, medical and environmental sciences	United States of America	EIDs
[38] Baum, Machalaba, Daszak, Salerno & Karesh	The authors conducted a review of OH literature to determine the current status of OH frameworks and case studies reporting OH metrics. They also reviewed possible outcome metrics suitable for the future evaluation of OH.	OH in the ID sense, with the integration of veterinary, medical and environmental sciences	Non-geographically located	The OH approach in itself
[2] Bidaisee & Macpherson	The overall objective was to determine the status of the OH approach and its applications to zoonotic diseases.	OH in the ID sense, with the integration of veterinary, medical and environmental sciences	Non-geographically located	Zoonosis
[10] Binot, Duboz, Promburom, Phimpraphai, Cappelle, Lajaunie, Goutard … Roger	A conceptual framework for OH integration is proposed. The authors suggest to develop a new culture of networking through a participatory modelling process to stimulate long-term dialogue process. It aims for implementing both institutional OH dynamics and research approaches promoting systems thinking and involving social sciences to follow-up and strengthen collective action.	OH in the ID sense and advocate for the adoption of the a TD/ participatory approach	Southeast Asia	Global health challenges
[20] Boischio, Sanchez, Orosz & Charron	This paper presents an overview of Ecohealth research approaches applied to vector-borne diseases, with particular attention to multi-stakeholder participation given its prominence in the sustainable development policy discourse.	Ecohealth in the TD sense, beyond academic disciplines	Tropical developing countries	Vector borne diseases
[57] Busani, Caprioli, Macri, Mantovani, Scavia & Seimenis	This paper discusses some problems nowadays facing the practice of collaboration as well as special issues concerning the Mediterranean area and Europe.	One Medicine in the multidisciplinary sense with the involvement of veterinary, medical, environmental actors	Mediterranean countries in Europe	Veterinary public health
[26] Buttke, Decker & Wild	This article explores the challenges and opportunities for addressing wildlife conservation in a OH context.	OH in the ID sense, with the integration of veterinary, medical and environmental sciences	Non-geographically located	EIDs and wildlife conservation
[34] Cars, Xiao, Lundborg, Nilsson, Shen, Sun … Wang	In this article, the authors describe a OH research project that addresses gaps in current knowledge through a cross-disciplinary collaboration called ‘The Sino-Swedish Integrated Multi-sectoral Partnership for Antibiotic Resistance Containment’ (IMPACT). The aim is also to contribute to the limited discourse on cross-country multi-sectoral collaborations in OH.	OH in the ID sense, with the integration of veterinary, medical and environmental sciences	China	Antimicrobial Resistance
[47] Conrad, Meek & Dumit	The advantages and challenges posed by OH ID collaborations are described in this review. The 11 papers in this special issue are also introduced as they illustrate how a OH approach can be applied to better understand and control zoonotic pathogens, engage community stakeholders in OH research and utilize wildlife species.	OH used in different levels of collaboration and integration	Non-geographically located	Zoonosis and wildlife
[54] Dorea, Sanchez & Revie	This paper reviews recent progress in the development of syndromic surveillance systems for veterinary medicine. Peer-reviewed and grey literature were searched in order to identify surveillance systems that explicitly address outbreak detection based on systematic monitoring of animal population data, in any phase of implementation.	One Medicine in the multidisciplinary sense with the involvement of veterinary and medical science	Developed countries	Veterinary surveillance
[28] Ducrotoy, Yahyaoui Azami, El Berbri, Bouslikhane, Fassi Fihri, Boue, Bardosh	In this paper it is presented an evaluation of an integrated health messaging intervention, for five bacterial and dog-associated zoonotic diseases. The aim is to encourage sequential adaptation of images, key messages, and delivery strategies using auto-evaluation and end-user feedback. The authors describe the challenges and opportunities of this approach. They conclude by discussing the merits of incorporating the validated education approach into the school curriculum in order to influence long-term behaviour change.	OH in the TD sense, beyond academic actors	Sidi Kacem Province, northwest Morocco	Neglected tropical diseases
[39] Durski, Jancloes, Chowdhary & Bertherat	The authors present the Global Leptospirosis Environmental Action Network (GLEAN) developed to improve global and local strategies of how to predict, prevent, detect, and intervene in leptospirosis outbreaks in order to prevent and control leptospirosis in high-risk populations.	One Medicine in the multidisciplinary sense with the involvement of actors from veterinary and medical sciences	Tropical developing countries	Zoonosis - Leptospirosis
[27] Ellis-Iversen, Cook, Watson, Nielen, Larkin, Wooldridge & Hogeveen	This study presents a theoretical framework from behavioural science, combined with basic epidemiological principles to investigate and explain the control of zoonotic agents on cattle farms.	OH in the TD sense, beyond academic actors	United Kingdom	Zoonosis
[40] Gebreyes, Dupouy-Camet, Newport, Oliveira, Schlesinger, Saif, … King	This article is a conference abstract and review to share and discuss issues related to zoonotic infectious diseases worldwide, by analysing scientific reports in eight thematic areas that necessitate OH implementation. The aim of this review is to highlight advances in key zoonotic disease areas and the OH capacity needs.	OH in the ID sense, with the integration of veterinary, medical and environmental sciences	Non-geographically located	Zoonosis
[18] Gongal	While there is a growing recognition of OH, it has to be translated from concept into actions through country level activities that are relevant for specific situations.	One Health in the multidisciplinary sense with the involvement of actors from veterinary, medical and environmental sciences	Southeast Asia	EIDs
[21] Hall & Le	The authors argue that for effective management of global health a basic strategy should include at least three essential tactical forms: actions of a directly focused nature, institutional coordination, and disciplinary integration in approaches to health management. Each level of action is illustrated with examples from the livestock sector in Asia.	OH in the ID sense, with the integration of veterinary, medical and environmental sciences	Asia	EIDs and livestock
[41] Harper, Edge & Cunsolo Willox	This article describes how an EcoHealth approach guided by principles of TD, community participation, and social equity was used to plan and implement a climate– health research project. An overview of the project, including project development, research methods, project outcomes to date, and challenges encountered, is presented.	Ecohealth in the TD sense, beyond academic actors	Canada	Environmental issues - climate change
[42] Häsler, Cornelsen, Bennani & Rushton	This paper explores the absence of adequate frameworks to measure OH benefits, with a review of the available literature and an examination of methods used. A framework for measuring the advantages of a OH approach is needed and, through the process of an international workshop and the development of a OH business case, the authors are working towards its development.	OH used in different levels of collaboration and integration	Non-geographically located	OH in itself
[59] Hinchliffe	This paper uses insights from Geography and Science and Technology Studies along with multi-sited and multi-species qualitative fieldwork on animal livestock and zoonotic influenzas in the UK, to highlight the importance of those practical engagements.	One world one health in the sense of TD collaboration with inclusion of socio-economic sciences	United Kingdom	Animal livestock and zoonotic influenzas
[19] Hung, Siobhan, Dinh Xuan, Mallee, Wilcox & Grace	The objective of this paper is to review Ecohealth activities within SEA over the last 10 years to address the lessons learned, challenges faced and the way forward for Ecohealth in the region.	Ecohealth in the TD sense, beyond academic actors	Southeast Asia	EIDs
[50] Hutchins, Brown & Poulsen	The authors defend that the influences shared by the human biology and social/cultural contexts must be made transparent for broadly targeted health initiatives, such as OH, to be successful. They then suggest that field experiences in culturally diverse settings can help students better understand the OH concept in a truly holistic sense. Finally, they share findings from a decade of students' reflections and survey data that can be useful for institutions trying to introduce cultural and social elements into the health sciences curricula.	OH in the ID sense with the inclusion of socio sciences	Ecuador	OH education
[43] Kayunze, Kiwara, Lyamuya, Kambarage, Rushton, Coker & Kock	The research from which this article emanated was done with the specific objectives to: (1) determine the proportions of health experts who had collaborated with other experts of disciplines different from theirs, (2) rank the general bridges for and barriers to collaboration according to the views of the health experts, and (3) find the actual bridges for and barriers to collaboration among the health experts interviewed.	OH in the ID sense with the inclusion of socio sciences	Tanzania	OH in itself
[60] Kingsley, Patrick, Horwitz, Parkes, Jenkins, Massy, … Arabena	This article highlights contributions that can be made to the public health field by incorporating “ecosystem approaches to health” to tackle future environmental and health challenges at a regional level. This qualitative research reviews attitudes and understandings of the relationship between public health and the environment and the priorities, aspirations and challenges of a newly established group (the Oceania EcoHealth Chapter) who are attempting to promote these principles.	Ecohealth in the TD sense, beyond academic actors	Oceania	Environmental issues
[29] Lam, Leffley, & Cole	The authors applied an Ecohealth perspective into a State of the Environment report for Grey Bruce Health Unit and summarized environmental and health data relevant for public health practice. This paper aims to (1) describe our efforts at adopting an Ecohealth approach to SOE reporting at the local county level; (2) present the framework we used for organizing environmental, ecosystem and health data; and (3) discuss the challenges encountered and options for future development.	Ecohealth in the TD sense, beyond academic actors	Canada	Environmental issues
[61] Lapinski, Funk & Moccia	Based on an ID collaboration, this paper reports on a nascent conceptual framework for the role of social science in OH issues and identifies a series of recommendations for research directions that bear additional scrutiny and development.	OH in the ID sense with the inclusion of socio sciences	Non-geographically located	OH in itself
[53] Leach, & Scoones	In this article the authors argue that OH challenges are best met by combining multiple models and modelling approaches that elucidate the various epidemiological, ecological and social processes at work.	OH in the ID sense with the inclusion of socio sciences	Developing countries	Zoonosis
[25] Lebov, Grieger, Womack … MacDonald	The authors discuss key steps in designing an OH study and illustrate these concepts through the presentation of a case study of health impacts associated with land application of biosolids. Finally, they discuss opportunities for applying an OH approach to identify solutions to current global health issues, and the need for cross-disciplinary funding sources to foster an OH approach to research.	OH in the ID sense with the inclusion of veterinary, medical and environmental sciences	Non-geographically located	OH in itself
[22] Lee & Brumme	This commentary explores the prospects of operationalizing the OH approach within current institutional arrangements that comprise global health governance.	OH in the ID sense with the inclusion of veterinary, medical and environmental sciences	Non-geographically located	OH in itself
[51] Lerner & Berg	The paper aims to discuss four topics under the OH umbrella: the demarcation of OH; the concept of health; practical consequences for research; and practical consequences for education.	Use of the different OH concepts	Sweden	OH in itself
[17] Leung, Middleton & Morrison	The purpose of this study was to characterize how public health actors in Ontario are influenced by the holistic principles, which underlie One Health and EcoHealth, and to identify important lessons from their experiences.	OH (as ID) and Ecohealth (as TD)	Ontario, Canada	Public health
[4] Min, Allen-Scott & Buntain	In this scoping review, the authors investigated key concepts, definitions and themes in One health and transdisciplinary research based on the peer reviewed literature.	OH in the TD sense, with the involvement of actors beyond the academic domain	Non-geographically located	OH in itself
[30] Morse	This article discusses public health surveillance and major recent surveillance initiatives and reviews progress towards implementing a OH surveillance framework.	OH in the ID sense with the inclusion of veterinary, medical and environmental sciences	Non-geographically located	Zoonosis
[58] Nguyen, Nguyen-Viet, Pham-Duc, Stephen & McEwen	The authors investigated a project of health and environmental sanitation to identify the impediments and enablers of Ecohealth and investigate how it can move from concept to practice.	Ecohealth in the TD sense, beyond academic actors	Hà Nam, Vietnam	Environmental issues
[31] Okello, Bardosh, Smith & Welburn	A qualitative case study methodology was used to examine the emerging relationships between international OH dialogue and its practical implementation in the African health policy context. Through the interviewing a selection of respondents currently at the forefront of policy development attempts have been made to address this issue.	OH in the ID sense with the inclusion of veterinary, medical and environmental sciences	Africa: Uganda, Nigeria and Tanzania.	Zoonosis
[55] Ope, Sonoiya, Kariuki, Mboera, Gandham, Schneidman & Kimura	The objective of this paper is to review the regional EAIDSNet initiative and highlight achievements and challenges in its implementation.	OH in the ID sense with the inclusion of veterinary, medical and environmental sciences	East Africa	Infectious disease surveillance
[9] Papadopoulos & Wilmera	This paper will provide an overview of OH, the evolution of the movement, and the current challenges. Also, this paper will showcase the application of OH through the Canadian response to West Nile virus and will conclude with recommendations and steps forward.	OH in the TD sense, with the involvement of actors beyond the academic domain	Canada	Emerging zoonotic diseases
[49] Pezzoli, Kozo, Ferran, Wooten, Gomez & Al-Delaimy	This article examines a bioregional effort to merge place-based health planning and ecological restoration along the US–Mexico border. The theoretical construct underpinning this effort is called One Bioregion/One Health (OBROH) and aims to improve trans-border knowledge networking, ecosystem resilience, community participation in science–society relations, leadership development and cross-disciplinary training.	One Bioregion/One Health in the TD sense, with involvement of actors beyond the academic domain	US–Mexico border	Bio regionalisation
[35] Schurer, Mosites, Li, Meschke & Rabinowitz	The authors conducted a systematic review of English and Chinese language peer-reviewed and grey literature and databases to identify zoonotic endoparasite research utilizing an OH approach in community-based settings. The aim is to identified important gaps in parasitology research operating under an OH framework and provide recommendations.	OH in the ID sense with the inclusion of veterinary, medical and environmental sciences	Non-geographically located	Zoonotic endoparasitosis
[44] Schurer, Phipps, Okemow, Beatch, & Jenkins	The authors report the effects of introducing a community action plan designed to improve animal and human health, increase animal health literacy and benefit community well-being in two Indigenous communities where a dog-related child fatality recently occurred.	OH in the TD sense, with the involvement of actors beyond the academic domain	Saskatchewan, Canada	Rabies
[23] Smith, Taylor & Kingsley	This paper traces the emergence and tensions of an internationally constructed and framed One World-One Health (OWOH) approach to control and attempt to eliminate African Trypanosomiasis in Uganda.	OH in the ID sense with the inclusion of veterinary, medical and environmental sciences	Uganda	African Trypanosomiasis
[52] Spiegel, Breilh, Beltran, Parra, Solis, Yassi, … Parkes	To assess progress in achieving desired outcomes in the Sustainably Managing Environmental Health Risk in Ecuador project, the authors reviewed results associated with the logic framework analysis used to guide the project, focusing on how a community of practice network has strengthened implementation, including follow-up tracking of program trainees and presentation of two specific case studies.	Ecohealth in the TD sense, beyond academic actors	Ecuador	Environmental issues
[45] Stark, Arroyo Kuribrena, Dauphin, Vokaty, Ward, Wieland & Lindberg	This paper is report of the second International Conference on Animal Health Surveillance (ICAHS) to discuss the relevance of OH in the context of surveillance. A number of success stories were presented and recommendations to advance the OH concept were provided.	OH in the ID sense with the inclusion of veterinary, medical and environmental sciences	Non-geographically located	Zoonotic surveillance
[48] Stephen & Stemshorn	This paper examines experiences in sectors other than OH, dealing with complex, cross and inter-sectoral problems. The objective is to advocate for increased investment in OH leadership, governance and partnership skills to balance the focus on creating cross-disciplinary awareness and technical proficiency in order to maintain OH as a viable approach to health issues at the human-animal-environment interface.	OH in the TD sense, with the involvement of actors beyond the academic domain	Non-geographically located	OH in itself
[56] Wallace, Bergmann, Kock, Gilbert, Hogerwerf, Wallace & Holmberg	In this critical review, the authors unpack the relationship between OH science and its political economy, particularly the conceptual and methodological trajectories by which it fails to incorporate social determinants of epizootic spillover. They also introduce a Structural OH that addresses this research gap.	OH in the TD sense, advocating for the involvement of social sciences	Non-geographically located	OH in itself
[5] Welburn	The author assesses recent progress in developing the OH concept, and where the challenges remain.	OH in the ID sense with the inclusion of veterinary, medical and environmental sciences	Non-geographically located	Emerging zoonotic diseases
[24] Wendt, Kreienbrock, & Campe	The authors aimed to provide an overview of existing systems throughout the world which integrate information from humans and animals on zoonotic diseases.	OH in the ID sense with the inclusion of veterinary, medical and environmental sciences	Non-geographically located	Zoonosis
[46] Willingham, Cruz-Martinez, Scorpio & Gallagher	This paper is a conference abstract about Caribbean regional challenges related to community-based approaches for zoonotic disease control and prevention, and mitigation of problems at the interface of wildlife, domestic animals, and humans. Participants suggested a framework for practicing OH in the Caribbean that emphasized capacity building and sustainability.	OH in the ID sense with the inclusion of veterinary, medical and environmental sciences	Caribbean countries	Zoonosis
[62] Wolf	This essay locates the one health discussion on disease ecologies in a more than human world within recent developments in cultural and medical anthropology that focus on the entanglements between health and a multitude of animals, plants or microbes, as they are characteristic of a globalized modernity. The paper aims to examine the social dimensions of human–animal-disease-interactions, claiming that disease is a biocultural phenomenon and that social factors generally play a crucial role in the emergence, spread and management of (infectious) disease.	OH in the TD sense, with the involvement of actors beyond the academic domain	Non-geographically located	OH in itself
[65] Yates-Doerr	This article, through its descriptions of the contingencies scientists face when producing edibility, suggests that “the global,” at least when it comes to food security, cannot be a singular thing and thus cannot be addressed by a universal approach or commonly shared solution.	One World One Health in the ID sense, with the involvement of human, animal and environmental sciences	Non-geographically located	Food security
[64] Zinsstag, Schelling, Waltner-Toews & Tanner	This paper firstly recalls briefly the history of integrative thinking on human and animal health, secondly it reviews “one medicine” and “ecosystem approaches to health” in the conceptual landscape of comparable and neighbouring approaches, and thirdly it explores avenues of systemic approaches to the health of animal and humans and their potential to address the challenges ahead.	OH in the TD sense, with the involvement of actors beyond the academic domain	Non-geographically located	OH in itself
